# Circumferential and Depth Variations in Tissue Dielectric Constant Values as Indices of Lower Leg Localized Skin Water

**DOI:** 10.7759/cureus.27617

**Published:** 2022-08-02

**Authors:** Harvey N Mayrovitz

**Affiliations:** 1 Medical Education, Nova Southeastern University Dr. Kiran C. Patel College of Allopathic Medicine, Davie, USA

**Keywords:** lymphedema detection, lymphedema measurement, tissue dielectric constant (tdc), lower extremity lymphedema, leg edema, leg lymphedema measurement, edema, lymphedema

## Abstract

The primary goal of this study was to quantify circumferential (medial, anterior, lateral) and measurement depth variations (0.5 mm, 1.5 mm, 2.5 mm) in tissue dielectric constant (TDC) values as an aid to their use to assess the presence and progression of lower extremity edema and lymphedema. Measurements were done in 30 healthy non-edematous women to provide reference data to estimate expected values and thresholds when evaluating clinical edematous or lymphedematous conditions. A second goal was to determine the extent to which TDC values evaluated at lower leg sites depend on body mass index (BMI). The study protocol (#12180901) was approved by the university’s institutional review board and subjects were evaluated after signing an approved informed consent. The study group had an age range of 19-54 years with a mean age and SD of 30.6 ± 10.1 years and had a BMI between 18.1-44.1 Kg/m^2^ with a mean BMI and SD of 24.5 ± 5.4 Kg/m^2^. The main findings show that at the three circumferential sites (medial, anterior, and lateral) located eight cm from the mid-malleolus, there are small but statistically significant differences in TDC values at every measurement depth (0.5 mm, 1.5 mm and 2.5 mm). For each depth, the maximum difference occurs between the medial and lateral locations with lateral locations having a greater TDC value at all depths. Despite the wide range of BMI values of the subjects evaluated, no significant relationship between TDC value and BMI was detected. It is concluded that TDC measurements in the lower leg reveal statistically significant differences among circumferential sites and measurement depths that should be considered when evaluating or tracking lower extremity tissue water changes associated with edema, lymphedema or other conditions related to skin water. The absolute values of these non-edematous TDC values herein determined may provide a basis for calculating TDC thresholds applicable to edematous or lymphedematous lower leg conditions.

## Introduction

Tissue dielectric constant (TDC) values may be used to measure skin water because TDC values are directly dependent on skin-to-fat water content [1-4]. Measurements are achieved by gently touching the skin with a probe for a few seconds to obtain the associated TDC value. One main use of this methodology has been to assess lymphedema status in women at risk for breast cancer treatment-related lymphedema (BCRL) [5-15]. TDC measurements have also been used to obtain information on leg skin water in healthy women [16,17] and in women with lower extremity edema or lymphedema [18-22]. When lower extremity TDC measurements are done on healthy persons, who are free of edema or lymphedema, the values depend on the leg location measured [17,19].

Jensen and co-workers measured TDC values on the foot dorsum, medial ankle, and lateral lower leg of 34 young women to a depth of 2.5 mm and found significant differences in TDC values among sites [16]. This is important information that also provides an indication of TDC value variations that may be expected at a given longitudinal leg location. Further important work was done by Jonsson et al. who measured TDC values, also to a depth of 2.5 mm, at multiple longitudinal leg sites at medial, lateral, and posterior circumferential sites [17]. In addition to knowing TDC circumferential variations at lower leg sites, another important aspect is the amount of variation when such measurements are made to different tissue depths. Prior work has shown that such depth variations are present in arms [23-25], but similar data is not readily available for lower extremities.

Thus, the primary goal of the present study was to characterize the circumferential variation in TDC values at a commonly evaluated lower leg site and evaluate the dependence of such variations on tissue depths to which measurements are made. Since clinical assessments of TDC values may be measured using probes that record to various depths, one importance of this investigation is that it provides insight into the relative contributions of the depth-dependent tissue components of the lower leg that affect TDC values. A second goal was to determine the extent to which these TDC measurements were dependent on body mass index (BMI). The motivation for this part of the investigation relates to the potential dependence of TDC values on relative fat content within the measured volume due to the low water content of fat tissue [26,27]. 

## Materials and methods

Subjects

Entry requirements for participation in this study were being female at least 18 years of age, having no extensive hair on the lower leg, being willing to refrain from putting lotions or creams on the legs on the day of measurement, and having no open sores in the areas of measurement. Exclusionary criteria were the presence of any open wound near an intended measurement site and the presence of any implanted wires or electronic medical devices. Participants were healthy women recruited from medical students and staff and evaluated after signing an informed consent approved by the university institutional review board (Protocol # 12180901). The inclusion of a single sex in this study diminishes potential confounding effects due to known differences in male vs. female TDC values [28-30]. In addition, although lymphedema affects both genders it is more prominent among women [31-33]. This paper reports on the 30 healthy women recruited for the study. They had an age range of 19-54 years with a mean and SD of 30.6 ± 10.1 years and had a body mass index (BMI) from 18.1-44.1 Kg/m^2^ with a mean and SD of 24.5 ± 5.4 Kg/m^2^.

TDC measurement

**Figure 1:**
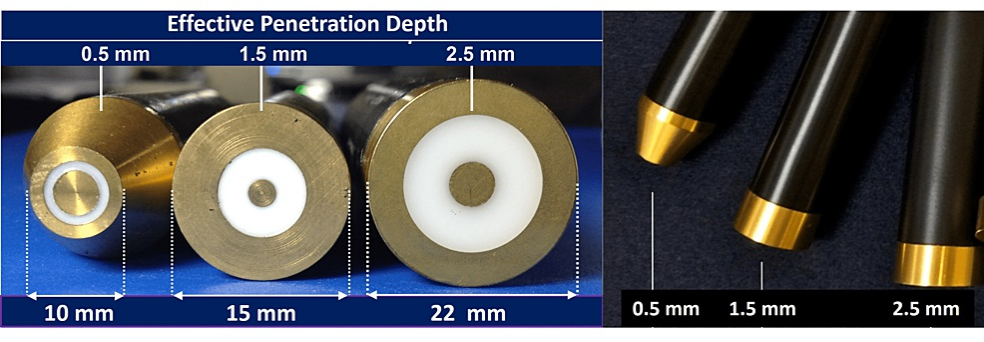
Probes Used to Measure Tissue Dielectric Constant (TDC) Diameters of the three probes used in the present study are shown in the left panel and their corresponding longitudinal images on the right panel. The center and outer conductors are separated by the white insulating material. The probes are connected to a control box though a coaxial cable. Touching the skin with the probe activates the measurement process that takes about 5-7 seconds whereupon the TDC value is displayed on the control box display.

The TDC measuring device (MoistureMeterD, Delfin Technologies, Kuopio, Finland), determines the dielectric constant (relative permittivity) that is a dimensionless number equal to the ratio of tissue permittivity to vacuum permittivity. Because TDC values mainly depend on tissue water they provide quantitative indices of skin water content. The measurement is sensitive to both free and bound water [34]. Inclusion of the bound water contribution is useful since up to 80-90% of adult skin water content is bound [35]. The device generates and transmits a low power 300 MHz signal into skin via an open-ended coaxial transmission line [4]. The reflected part of the signal allows calculation of the complex reﬂection coefﬁcient from which TDC is determined [1]. Reﬂections depend on the tissue’s dielectric constant (real part of its complex permittivity), its conductivity and the frequency. At 300 MHz, conductivity contributes little to the overall permittivity value and TDC is mainly determined by free and bound water molecules. More details including validation data are described in the literature [3]. Three different probes were used in this study to measure to effective depths of 0.5 mm, 1.5 mm, and 2.5 mm (Figure 1). A probe is coupled to a control box via a coaxial cable and placement of a probe in contact with skin automatically activates the measurement. After about five seconds the TDC value is displayed on the device’s control box. Each probe is calibrated against various ethanol-water mixture concentrations each of known dielectric constant values [36].

Procedures

**Figure 2:**
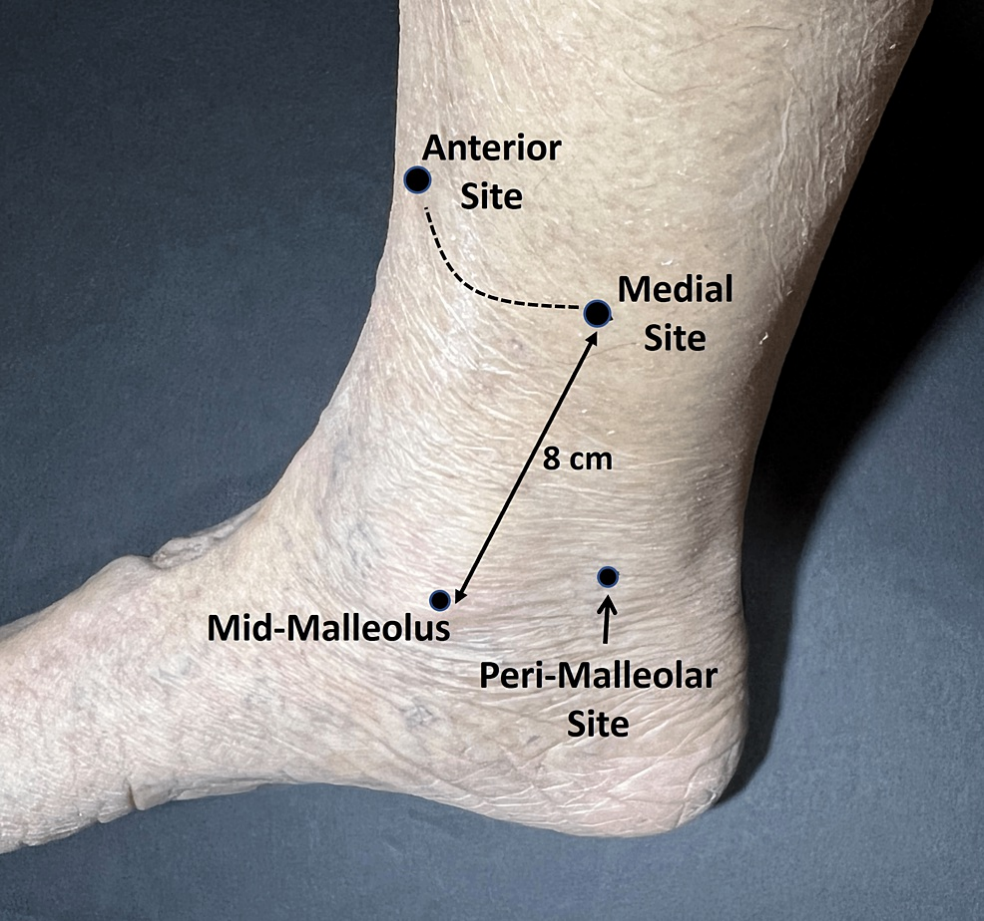
Lower leg TDC measurement sites TDC was measured at the indicated sites and the lateral site (not shown) The three circumferential sites were located 8 cm proximal to the mid-malleolus whereas the peri-malleolar site was located three cm below the mid-malleolar point. The order of the measurements was peri-malleolar, medial, anterior, and lateral respectively. After completion of the 2.5 mm depth measurement sequence, it was done with the 1.5 mm depth probe and finally done with the 0.5 mm depth probe.

With the subject supine on a padded examining table, a site on the medial, anterior, and lateral aspects of the right leg located eight cm proximal to the mid-malleolus were marked with a skin marker (Figure 2). In addition, a peri-malleolar site was marked that was located three cm below the mid-malleolar point as illustrated in Figure 2. These sites were the target TDC measurement sites because of they are often used to assess the presence of lower extremity. After a supine acclimation interval of 10 minutes, TDC was measured at each of the four sites once starting with the 2.5 mm depth probe. The order of the measurements was peri-malleolar, medial, anterior, and lateral respectively. After completion of the 2.5 mm depth measurement sequence the same measurement sequence was done with the 1.5 mm depth probe and finally done using the 0.5 mm depth probe. After completing all TDC measurements the skin temperature at each site was measured using an infrared thermometer (Model DX501, Exergen, Watertown MA, USA).

Analyses

Tests for overall differences among sites (medial, anterior, lateral, peri-malleolar) and among depths (0.5 mm, 1.5 mm, and 2.5 mm) at a given circumferential site were tested using a general linear model with repeated measures. A statistically significant overall difference among depths or sites was based on a p-value < 0.01. Regression analysis was used to test for a possible relationship between TDC and BMI.

## Results

Table 1 summarizes TDC values obtained for measurements at medial, anterior, lateral, and peri-malleolar lower leg sites for each of the three measurement depths. Also shown in Table 1 are the skin temperatures at each site.

**Table 1:** TDC values by site and Depth Table entries are tissue dielectric constant (TDC) values (mean ± SD) for each measured site and depth and skin temperature measured at these sites. Except for the peri-malleolar site, TDC values decreased (p<0.001) with increasing measurement depth at each site. At each measurement depth, TDC values differed among sites (p<0.001). The temperature at the lateral site was slightly but statistically greater than all other sites (*) p<0.01.

	TDC Measurement Site			
Depth (mm)	Medial	Anterior	Lateral	Peri-malleolar
0.5	32.5 ± 5.2	34.3 ± 4.4	35.5 ± 4.7	26.6 ± 4.1
1.5	31.1 ± 5.9	32.5 ± 3.9	33.6 ± 3.8	26.4 ± 3.3
2.5	28.0 ± 4.6	30.1 ± 4.2	33.0 ± 5.3	26.3 ± 3.2
Skin Temp (^o^C)	31.3 ± 1.1	31.4 ± 1.1	31.9 ± 1.6*	30.8 ± 1.6

TDC variations by depth

TDC values decreased with increasing measurement depth at each site (p<0.001) except at the peri-malleolar site. This site had the lowest TDC values of any site with mean values ranging from 26.6 at a depth of 0.5 mm to 26.3 at a depth of 2.5 mm. The site with the greatest mean values was the lateral site with values ranging from 35.5 at a depth of 0.5 mm to 33.0 at a depth of 2.5 mm. Considering the mean percentage difference in TDC values between 0.5 mm and 2.5 mm depths indicates that the TDC value at a depth of 2.5 mm is less than that of the 0.5 mm value by 16.1% at the medial site, 14.0% at the anterior site and 7.6% at the lateral site. 

**Figure 3:**
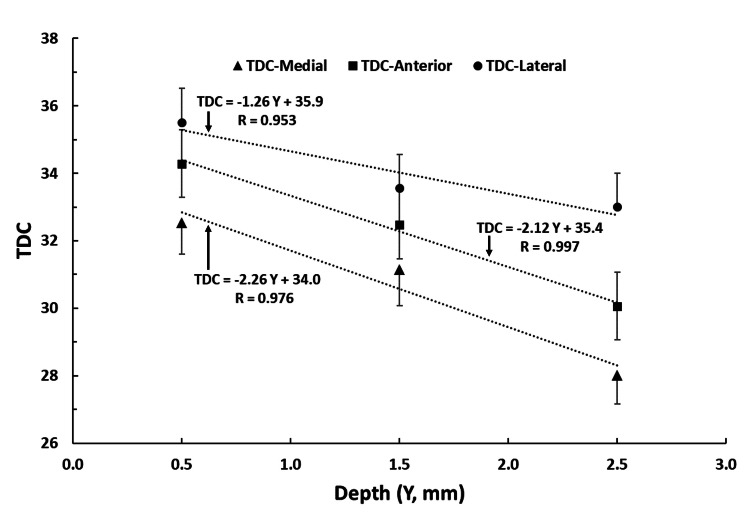
TDC variations with measurement depth and circumferential site Data points are mean values for N =30 subjects and error bars are the standard error of the mean (SEM). Equations are linear regression of tissue dielectric constant (TDC) value vs. measurement depth (Y) for lateral (circles), anterior (squares) and medial (triangles) sites. R values are the sample correlation coefficients. All regressions are statistically significant (p<0.001).

Figure 3 graphically shows the depth variation for each circumferential site (medial, anterior, and lateral) with an associated linear regression line showing the statistical significance of TDC-Depth dependence for these lower leg sites. A similar depth dependence pattern has been shown to be present on the forearm [37,38] and has been explained to occur due to increasing amounts of low water content fat included in the measurement volume with increasing depth [39].

TDC variations by site

The TDC variations among circumferential sites summarized in Table 1 also show that at all measurement depths there is a progressive increase (p<0.001) in TDC values from medial to anterior to lateral sites. The largest mean percentage difference (17.9%) is between medial and lateral sites when measured to a depth of 2.5 mm. When these sites are measured to a depth of 0.5 mm this difference is reduced to 9.2 %.

TDC variations by BMI

**Figure 4:**
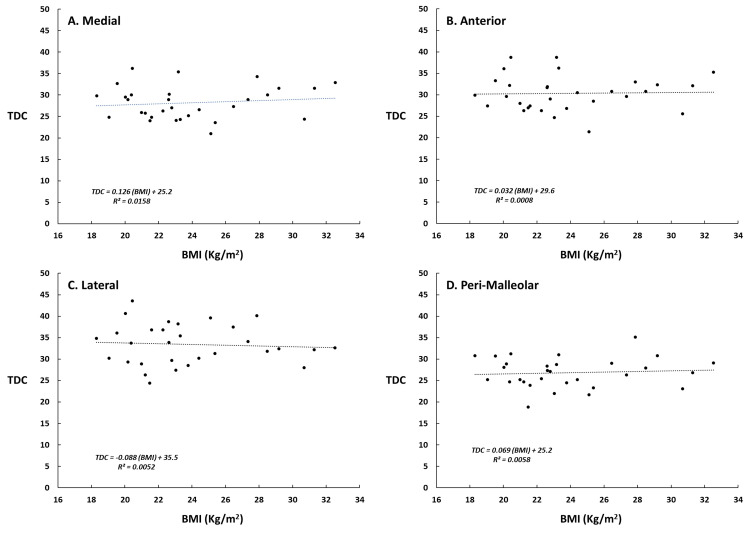
TDC variations with subject’s BMI Data points are individual subject tissue dielectric constant (TDC) values measured at each of the four lower leg sites to a depth of 2.5 mm. The superimposed line is the calculated regression line. As may be noted from the regression equations and R values there was no significant relation between TDC value and BMI at any of the measured sites in this group of subjects.

Attempts to find a significant relationship between TDC values and a subject’s BMI using regression analysis were not successful as demonstrated graphically in Figure 4 for measurements to a depth of 2.5 mm. Despite the reasonably wide BMI range of the studied subjects no significant relationship was demonstrated at any of the sites for any measurement depth.

## Discussion

The focus of the present study was to provide quantitative information on normal lower leg TDC values, specifically their variation with circumferential measurement site, measurement depth, and possible dependence on BMI. The purpose was to provide initial guidance as to variations among healthy women that could subsequently serve as a reference for evaluating women with lower extremity edema or lymphedema. The main findings show that at the three circumferential sites (medial, anterior, and lateral) located eight cm from the mid-malleolus, there are small but statistically significant differences in TDC values at every measurement depth (0.5 mm, 1.5 mm, and 2.5 mm). For each depth, the maximum difference occurs between the medial and lateral location with the lateral location having the greater TDC value at all depths. Despite the wide range of BMI values of the subjects evaluated, no relationship between TDC value and BMI was detected. This might be explained by the absence of a connection between the BMI and the amount of subcutaneous fat present at the lower leg sites measured. Future studies targeting this aspect should consider measuring leg fat percentages along with TDC measurements.

The observed TDC differences may be explainable based on differences in relative amounts of subcutaneous fat at these locations. The medial target site tends to contain more fat than the anterior or lateral sites within the depth of the TDC measurement. Given the low water holding capacity of fat, it is likely that the highest TDC values are at sites with lower fat content. The difference in TDC values among sites was found to be greatest when TDC was measured to a depth of 2.5 mm and least when it was measured to a depth of 0.5 mm. A possible explanation for this finding is also related to the tissue content included at varying measurement depths. At a depth of 0.5 mm, the TDC measurement includes contributions from the epidermis and part or all of the dermis, with the dermis having among the highest water concentration of the skin-to-fat components [35,40,41]. As measurement depth increases, the TDC value depends on increasing contributions of hypodermal fat. Hence the shallowest depth measurement has the highest TDC value as is shown by the current data set. 

Although these considerations may help explain variations observed among sites and depths in the lower leg region evaluated, the question remains as to their practical impact. One aspect seems to be clear, when using TDC measurements in patients to detect, track or evaluate treatment effects in the lower leg, careful referencing of initial measurement sites is strongly indicated with repeat measurements as close to the original as dictated by practicality. Also, although probably obvious, measurements should always be made to the same depth.

The present findings also add to our understanding of the range of variability in lower leg TDC values that may be expected. One useful aspect of this quantitation is to aid in judging normal values from those that may represent edematous or lymphedematous conditions. One approach to developing such a threshold is to consider TDC as abnormally high if TDC values exceed the mean value determined in the healthy population by two (or more) standard deviations (SD). This approach has been used to develop TDC thresholds for upper limb unilateral lymphedema using inter-arm TDC ratios of about 1.26 [13,42]. Based on TDC measurements taken in both lower legs of a small group of healthy women, an inter-leg 3SD threshold of 1.20 has been reported [43]. However, in many cases of lower extremity lymphedema, the condition is bilateral so inter-leg threshold ratios may not be useful whereas absolute values of TDC may be reasonable indicators of lymphedema presence or extent. Based on the data in Table 1 such threshold values may be calculated. For example, using a 2SD threshold for a measurement at the medial site to a depth of 2.5 mm yields a TDC threshold of 37.2 (28.0 + 2 x 4.6). Thus, a measured TDC value of greater than 37.2 implies the presence of significant edema or lymphedema. Other thresholds for other sites and depths can be calculated similarly using Table 1.

There are two limitations of the present study that should be considered. Firstly, the data and findings strictly apply to women. Because of previously reported differences between male and female TDC values at other anatomical sites, male values at the lower leg sites are likely to be greater in males [28,44]. Future work using similar measurements in men for comparison would be warranted. Secondly, the data and calculated thresholds are based on values obtained in non-lymphedematous legs. Thus, the threshold values herein determined are as yet unproven and should be tested against measurements in persons with demonstrated lower leg edema or lymphedema.

## Conclusions

TDC measurements in the lower leg reveal statistically significant differences among circumferential sites and measurement depths that should be considered when evaluating or tracking lower extremity tissue water changes associated with edema, lymphedema, or other conditions related to skin water. The absolute values of these non-edematous TDC values herein determined can provide a basis for calculating TDC thresholds applicable to edematous or lymphedematous lower leg conditions.
